# Sex-Related Responses of *Populus cathayana* Shoots and Roots to AM Fungi and Drought Stress

**DOI:** 10.1371/journal.pone.0128841

**Published:** 2015-06-23

**Authors:** Zhen Li, Na Wu, Ting Liu, Hui Chen, Ming Tang

**Affiliations:** 1 College of Life Science, Northwest A&F University, Yangling, China; 2 College of Forestry, Northwest A&F University, Yangling, China; Institute of Genetics and Developmental Biology, Chinese Academy of Sciences, CHINA

## Abstract

We investigated the impact of drought and arbuscular mycorrhizal (AM) fungi on the morphological structure and physiological function of shoots and roots of male and female seedlings of the dioecious plant *Populus cathayana* Rehder. Pot-grown seedlings were subjected to well watered or water-limiting conditions (drought) and were grown in soil that was either inoculated or not inoculated with the AM fungus *Rhizophagus intraradices*. No significant differences were found in the infection rates between the two sexes. Drought decreased root and shoot growth, biomass and root morphological characteristics, whereas superoxide radical (O_2_–) and hydrogen peroxide content, peroxidase (POD) activity, malondialdehyde (MDA) concentration and proline content were significantly enhanced in both sexes. Male plants that formed an AM fungal symbiosis showed a significant increase in shoot and root morphological growth, increased proline content of leaves and roots, and increased POD activity in roots under both watering regimes; however, MDA concentration in the roots decreased. By contrast, AM fungi either had no effect or a slight negative effect on the shoot and root growth of female plants, with lower root biomass, total biomass and root/shoot ration under drought. In females, MDA concentration increased in leaves and roots under both watering regimes, and the proline content and POD activity of roots increased under drought conditions; however, POD activity significantly decreased under well-watered conditions. These findings suggest that AM fungi enhanced the tolerance of male plants to drought by improving shoot and root growth, biomass and the antioxidant system. Further investigation is needed to unravel the complex effects of AM fungi on the growth and antioxidant system of female plants.

## Introduction

Poplar is a widely cultivated forest tree of high economic value [1]. However, poplars have a high level of water consumption and are drought-sensitive [2,3]. *Populus cathayana* Rehder, a typical dioecious plant, is an important ecological species that has spread widely in Qinghai province, China, which is an area that has suffered serious ecological degradation. Differences have been widely observed in dioecious plants between the plants that bear male flowers and the plants that bear female flowers in terms of morphology, physiology and ecology owing to their gender specialization [4,5,6,7]. For example, Han et al. [5] found male *P*. *cathayana* showed better self-protection of the photosynthesis system, higher contents of osmotic adjustment substances, and a better enzymatic detoxification cycle under drought than do females. Li et al. [7] also suggested that male *P*. *cathayana* showed a better water status, protection of membrance system, photosynthesis and chlorophyII fluorescence system under water stress than do females.

Various environmental stresses, such as salinity, ultraviolet radiation, and drought, severely affect the growth of *P*. *cathayana* [3,8,9]. Drought is considered to be the most serious abiotic stress, and seriously limits plant growth and productivity in arid and semiarid areas [10]. Water status is associated with biomass production and drought has been shown to significantly limit the growth of both shoots and roots [11]. Furthermore, when plants are stressed, reactive oxygen species (ROS) accumulate. Continuous ROS accumulation creates cytotoxic conditions [12]. To minimize the damage, plants have evolved various enzymatic antioxidants, such as superoxide dismutase (SOD), catalase (CAT) and peroxidase (POD) [13]. Generally, malondialdehyde (MDA) formation is considered to be an indicator of the extent of lipid peroxidation caused by oxidative stress [14]. However, powerful ROS scavenging systems, such as POD, SOD, and peroxidase (POX) activity, are stimulated, particularly in drought resistant plant. Proline, which has the same function, accumulates in response to water stress in higher plants and is known as an osmolyte for plant osmotic adjustment [15]. About 80% of terrestrial plants, including *P*. *cathayana*, are able to establish a symbiosis with arbuscular mycorrhizal (AM) fungi, which increases their capacity to tolerate drought [16]. Under most harsh environmental conditions, AM fungal symbioses are known as bioenhancers, and many field and pot experiments have shown that plants that have formed a symbiosis with an AM fungus are well adapted to water-deficit conditions [11,17]. Woody species that form an AM fungal symbiosis have been shown to be markedly more resistant to drought stress [18,19]. This is because the formation of an AM fungal symbiosis can, for example, improve water uptake by improving root growth, stomatal responses, and increasing P uptake [18], promote photosynthetic efficiency, enhance the antioxidant system and adjust the osmotic balance of the plant [20]. Transportation of water to the plant via fungal hyphae has also been suggested [21]. Furthermore, AM fungi had the additional advantages of increasing growth, yield [22], and nutrient acquisition by the host [23], as well as improving the soil structure owing to the production of glomalin by the AM fungus [24]. Different AM fungi have different effects on hosts [17]. Lu et al. [25] studied the effects of AM fungi on salt tolerance by *Populus tomentosa* males and females: although they found that AM fungal symbiosis had a positive effect to some extent, and they also reported complex effects on both sexes, such as enhancement in growth and salt tolerance.

The different responses of male and female *P*. *cathayana* trees to drought stress and the effects of AM fungal symbiosis on the tolerance of the trees to drought have been well documented. However, to date, there has been little investigation of the effects of AM fungal symbiosis on *P*. *cathayana* males and females that are under drought stress. The aim of this study was to compare the growth, morphology characteristics, and the antioxidant system of the shoots and roots of non-mycorrhizal and *Rhizophagus intraradics*-inoculated *P*. *cathayana* seedlings of both sexes under well-watered and water-limiting conditions and to examine the impact of AM fungi on the different sexes of this dioecious plant.

## Materials and Methods

### Plant and soil treatment

Cuttings of *P*. *cathayana*, 18 cm in length and 1.2 cm in diameter, were collected from 120 different trees (120 genotypes: 60 of each sex) that were sampled from 15 populations (eight adult trees at the same age stage per population), in a plant nursery in Sining, Qinghai Province, China. The owner of the nursery gave permission to conduct the study on this site. The cuttings were disinfected with 70% (v/v) ethanol for 15 s and then rinsed three times in sterile deionized water. Topsoil (0–20 cm) was collected from a field in which poplars were being grown in Yangling, Shaanxi Province, China, and sieved through a 2-mm sieve to provide a soil substrate for this study. The soil physicochemical properties were as follows: pH, 7.6 (measured in soil:water using a 1:5 ratio); available N, 37.42 mg/kg; available P, 12.34 mg/kg; available K, 134.50 g/kg; and organic matter, 18.88 g/kg. The soil substrate was then mixed with fine sand (v:v = 1:1) and autoclaved under pressure (0.11 MPa) at 121°C for 2 h.

### AM fungus inoculum

Inoculum of the AM fungus *Rhizophagus intraradics* JJ291 (BEG accession 158 at the International Bank for the Glomeromycota; http://www.hent.ac.uk/bio/beg/) consisted of spores (spore density was about 50 per gram of inoculant), mycelia, root fragments and soil.

### Experimental design

The 120 cuttings were planted in 4.5 l plastic pots filled with 4 kg of preconditioned soil matrix and grown in the greenhouse at 25–30°C with 12 h light per day. The experimental layout included three factors: sex (male or female), inoculation status (inoculated or not inoculated) and water regime (well watered or drought). Sixty pots (30 containing male cuttings and 30 containing female cuttings) were inoculated with AM fungus inoculum (20 g/pot), and the remaining pots (controls) were inoculated with 20 g of autoclaved inoculum with 10 ml of inoculum washing solution that had been filtered through a 1-μm nylon mesh to remove the live inoculum. Pots were arranged in a randomized complete block design. All the pots were initially well watered and kept at 85%–90% of field capacity. After 50 days growth the pots containing the 30 males and 30 females that had received the inoculation treatment were each divided into two groups of 15. The 15 inoculated pots containing males and 15 inoculated pots containing females that were to be subjected to the drought treatment were left unwatered until the soil reached 25%–30% field capacity. The rest of the inoculated pots received the well-watered treatment (controls) and were kept well watered (85%–90% of soil field capacity). All pots were kept at a stable field capacity for 30 days. Throughout the experiment, all pots were weighed and watered every day at 16:00 h to maintain the experimental soil field capacity. Six seedlings of each sex and treatment were randomly selected to measure the root infection rate, growth, root morphology, superoxide radical (O_2_^−^) and hydrogen peroxide (H_2_O_2_) content, POD activity, MDA concentration, and proline content.

### Root infection rate measurement

Samples of the fresh roots were collected immediately after the seedlings were harvested, gently washed, cut into 1-cm pieces, and fixed with FAA solution. 10% KOH and 0.05% trypan blue in lactophenol were used to clear and stain the root samples [26]. Root colonization was examined under the microscope and evaluated as described by Giovanetti and Mosse [27]. Data were recorded as the proportion of colonized root length. The presence or absence of AM fungi in the roots was further confirmed by performing a nested-PCR using the primer pairs NS5/ITS4 and GLOM1310/ITS4i, as described by Redecker [28].

### Growth measurement

Stem length and above-ground diameter were measured by tape and vemier caliper at the start and end of the experiment. And the growth of steam lengh (GSL) and growth of ground diameter (GGD) were calculated by day. The chlorophyll content (soil and plant analyzer development (SPAD) value) was measured at the end of the experiment with a chlorophyll meter (SPAD-502 Plus, Konica-Minolta Holdings, Inc., Osaka, Japan). At the end of the experiment, all the seedlings were harvested and divided into leaves, stem and roots. The leaf area (LA) was determined using coordinate paper. Total fresh weight of each part was weighed, and part in each sample was weighed, dried at 70°C for 48 h to constant weight and then weighed for water content measurement. The total biomass was calculated by subtracting the water content from the entire weight.

### Root morphology measurement

Six replicates of each sex and treatment were selected and the roots were carefully washed. Root length (RL), root volume (RV), root surface area (RSA), root tips number (RTN) and root average diameter (RAD) were measured with a WinRHIZO Root Analyzer System (WinRHIZO 2012b, Regent Instruments Canada Inc., Montreal, Canada) as described by Flavel et al. [29]. The roots were scanned by the Epson perfection V700 photo scanner (Seiko Epson Corp., Nagano, Japan) using the following parameters: default calibration method, Intrinsic; acquisition parameters, resolution medium 400 with image grey levels.

### Superoxide radical (O_2_^−^) and hydrogen peroxide (H_2_O_2_) content measurements

The O_2_^−^and H_2_O_2_ content was measured following the method of Zhang et al. [30]. First, 1-ml of hydroxylamine hydrochloride was added to the samples and left to react for 1 h. Then 1-ml of p-aminobenzene sulfonic acid and 1-ml of α-naphthylamine were added and kept at 25°C for 20 min before spectrophotometric analysis at 530 nm using NaNO_2_ as a standard curve. The H_2_O_2_ content was measured by forming a H_2_O_2_–titanium complex, which resulted from the reaction of tissue H_2_O_2_ with titanium tetrachloride. A sample buffer of 10 mM ascorbic acid was used as a control or blank for measuring H_2_O_2_ content.

### POD activity, malondialdehyde (MDA) concentration and proline content determination

The fully expanded leaves and roots of six plants of each treatment were selected randomly to analyze the POD activity, MDA and proline content. The samples were homogenized in sodium phosphate buffer (50 mM, pH 7.0), centrifuged at 10000 × *g* at 4°C for 10 minutes, and the supernatant of enzyme solution was collected for POD activity determination. The reaction was performed in a 3-ml solution comprising 10 μl of enzyme solution and 2.99 ml of sodium phosphate buffer (50 mM, pH 6.0) containing 18.2 mM guaiacol and 4.4 mM H_2_O_2_ as substrates. Peroxidase activity was expressed as the amount of enzyme required to change the optical density by 0.001 per minute at 470 nm and 25°C [31].

The MDA content was measured using a spectrophotometer (UV-2550, Shimadzu Co. Ltd., Japan) to determine the absorbance of the supernatant at 450, 532 and 600 nm as described by Kramer et al. [10]. The formula C (μM) = 6.45 (OD_532_—OD_600_)– 0.56 OD_450_ was used to calculate the MDA content.

The proline content was determined as described by Bates et al. [32]. Samples of 0.5 g of leaves and roots were homogenized in 10 ml of 3% sulfosalicylic acid solution and centrifuged at 10000 × *g* for 5 minutes. The supernatant was collected and 2 ml was mixed with 2 mg of ninhydrin reagent and 2 ml of pure acetic acid that had been heated in a water bath at 100°C for 1 h and cooled in an ice bath. Next, 4 ml of toluene was added and the mixture was shaken well for 20 s. The upper layer was collected and its absorbance at 520 nm was measured. Proline concentration was determined by using a standard curve (range 0–50 mg/ml).

### Statistical analysis

Experimental data were subjected to two-way and three-way analyses of variance (ANOVAs) and correlation analysis using the statistical software package SPSS 17.0 (SPSS Inc., Chicago, IL, USA). The means were compared by Duncan's multiple-range tests (*P*≤0.05) in a two-way and three-way ANOVAs. Two-way ANOVAs were used to evaluate the significance of drought, the inoculation treatment and their interaction in male and female plants. Three-way ANOVAs were performed to determine the significance of the effect of sex, the interaction of drought × inoculated treatment, drought × sex, inoculated treatment × sex, and sex × drought × inoculated treatment. The correlation analyses were tested by Pearson correlation coefficients.

## Results

### Inoculation and growth

**Fig 1:**
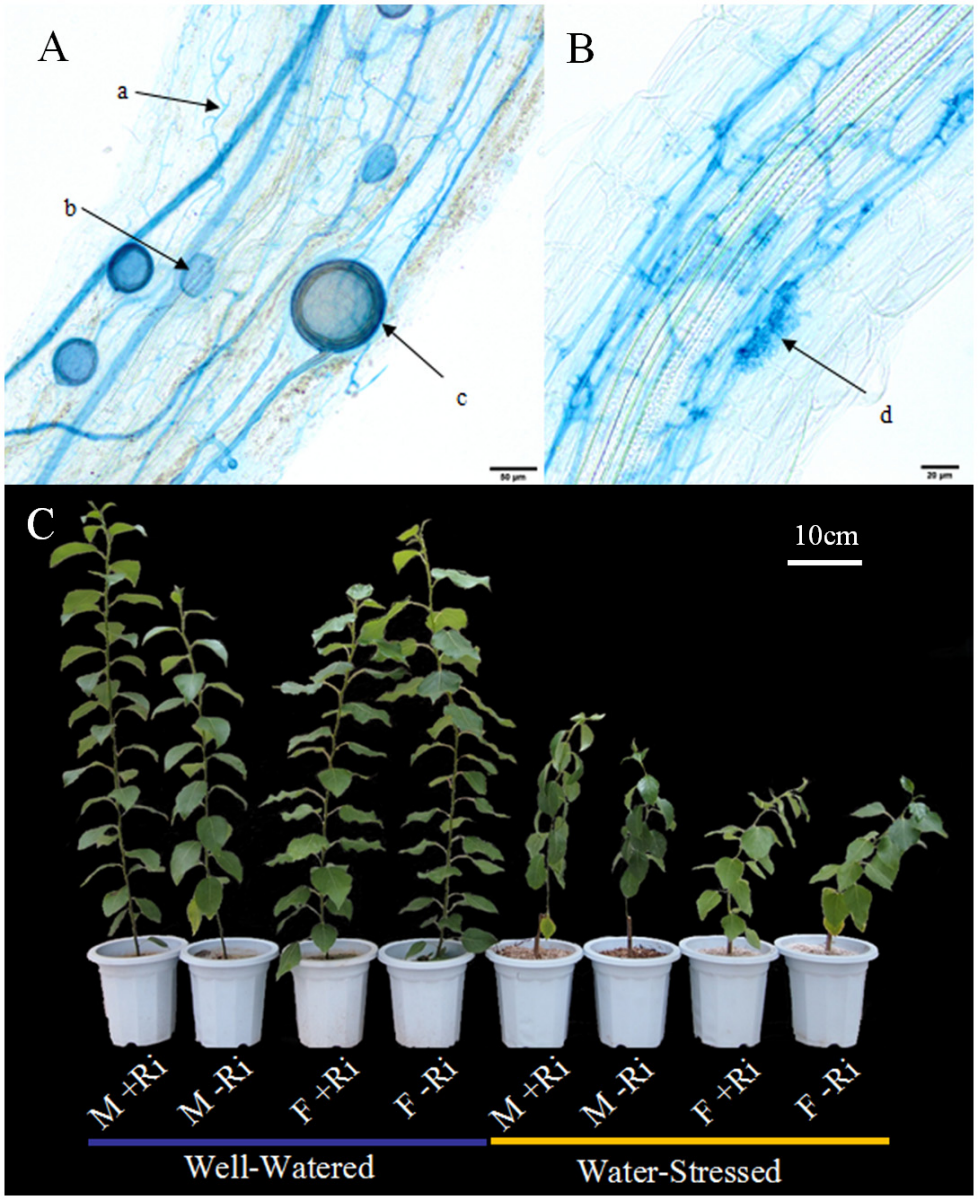
AM fungi structures in *P*. *cathayana* roots (A, B) and performance of the poplar seedlings with different treatment (C). a, hypha; b, vesicle; c, spore; d, arbuscule.

Both male and female poplars grown in pots inoculated with *R*. *intraradices* formed typical AM fungal structures (Fig 1A and 1B), whereas seedlings grown in pots that were not inoculated did not form any mycorrhizas. The absence of AM fungi in the roots of poplars that did not receive the inoculum treatments was further confirmed by performing a nested-PCR (data not shown). Apart from significantly higher hypha colonization rate under drought compared to well-watered conditions, vesicle, arbuscule and hypha infection rates showed no significant differences between sexes and drought treatments (Table 1).

**Table 1:** Infection rates of *P*. *cathayana* males and females under different watering regimes.

Treatments	Infection rate (%)			
	Vesicle	Arbuscule	Hypha	Total
AM M W	27.14±9.07	24.52±8.09	77.61±4.02^b^	86.80±3.06^b^
AM F W	35.80±20.70	34.78±14.03	84.73±5.29^ab^	87.19±3.22^b^
AM M D	32.18±3.95	51.29±20.60	92.60±5.30^a^	95.54±2.21^a^
AM F D	26.52±7.74	36.89±7.06	88.06±4.90^ab^	95.05±6.94^a^

**Abbreviations**: AM M W: AM fungi inoculation, male, well-watered; AM F W: AM fungi inoculation, female, well-watered; AM M D: AM fungi inoculation, male, drought; AM F D: AM fungi inoculation, female, drought. **Note**: The data are means ± SD (n = 6). Different lowercase letters (a, b) indicate a significant difference at *P* ≤ 0.05.

Both sexes were affected by water deficit and showed significantly reduced GSL, GGD, SPAD and LA compared with well-watered seedlings (Table 2). Male and female seedlings differed in their response to AM fungal formation. Male seedlings that had received the AM fungi inoculation treatment showed significantly increased LA compared under both water treatments and higher GSL under well-watered treatment with non-inoculated males. By contrast, the GSL, GGD, SPAD and LA of inoculated females were not significantly different with that of non-inoculated seedlings under both drought and well-watered conditions (Table 2, Fig 1C). Compared with non-inoculated seedlings, the inoculated males that received the well-watered treatment had significantly increased GSL, GGD and LA, as well as significantly decreased SPAD and LA under the drought treatment. The GGD and SPAD were significantly greater in males compared with females but LA was significantly smaller in males compared with the females in all treatments.

**Table 2:** Effects of AM fungal formation on growth parameters of *P*. *cathayana* males and females under different watering regimes.

Treatments			GSL (cm/d)	GGD (10^–2^ mm/d)	SPAD	LA (cm^2^)
Male	W	+M	1.02±0.14^a^	7.53±1.39^a^	44.73±1.42^a^	21.06±2.17^a^
		–M	0.58±0.10^b^	6.61±1.19^a^	43.57±1.17^a^	16.27±1.76^bc^
	D	+M	0.35±0.07^c^	4.57±1.41^b^	42.75±1.17^a^	17.79±1.22^b^
		–M	0.30±0.07^c^	3.36±0.60^b^	38.25±3.88^b^	14.05±3.84^c^
*P*_drought_			**	**	**	*
*P*_AMF_			**	*	**	**
*P*_drought×AMF_			**	NS	NS	NS
Female	W	+M	0.84±0.24^a^	5.76±1.01^a^	44.20±3.26^a^	25.77±8.09^a^
		–M	0.93±0.20^a^	5.86±0.65^a^	43.53±3.14^a^	22.67±5.51^ab^
	D	+M	0.23±0.20^b^	1.25±0.50^b^	35.82±5.25^b^	18.91±2.06^b^
		–M	0.25±0.02^b^	1.29±0.32^b^	35.50±4.368^b^	17.60±1.18^b^
*P*_drought_			**	**	**	**
*P*_AMF_			NS	NS	NS	NS
*P*_drought×AMF_			NS	NS	NS	NS
*P*_sex_			NS	**	**	**
*P*_drought×sex_			NS	*	*	NS
*P*_AMF×sex_			**	*	NS	NS
*P*_drought×sex×AMF_			*	NS	NS	NS

**Abbreviations**: +M: AM fungi inoculation;—M: non-inoculation; W: well-watered; D: drought; AMF: AM fungi.

**Note**:

*: significant effect at 0.01≤ *P* ≤ 0.05;

**: significant effect at *P* ≤ 0.01; NS: no significant effect.

The data are means ± SD (n = 6). Different lowercase letters (a, b, c, d) indicate a significant difference at *P* ≤ 0.05

### Biomass

**Fig 2:**
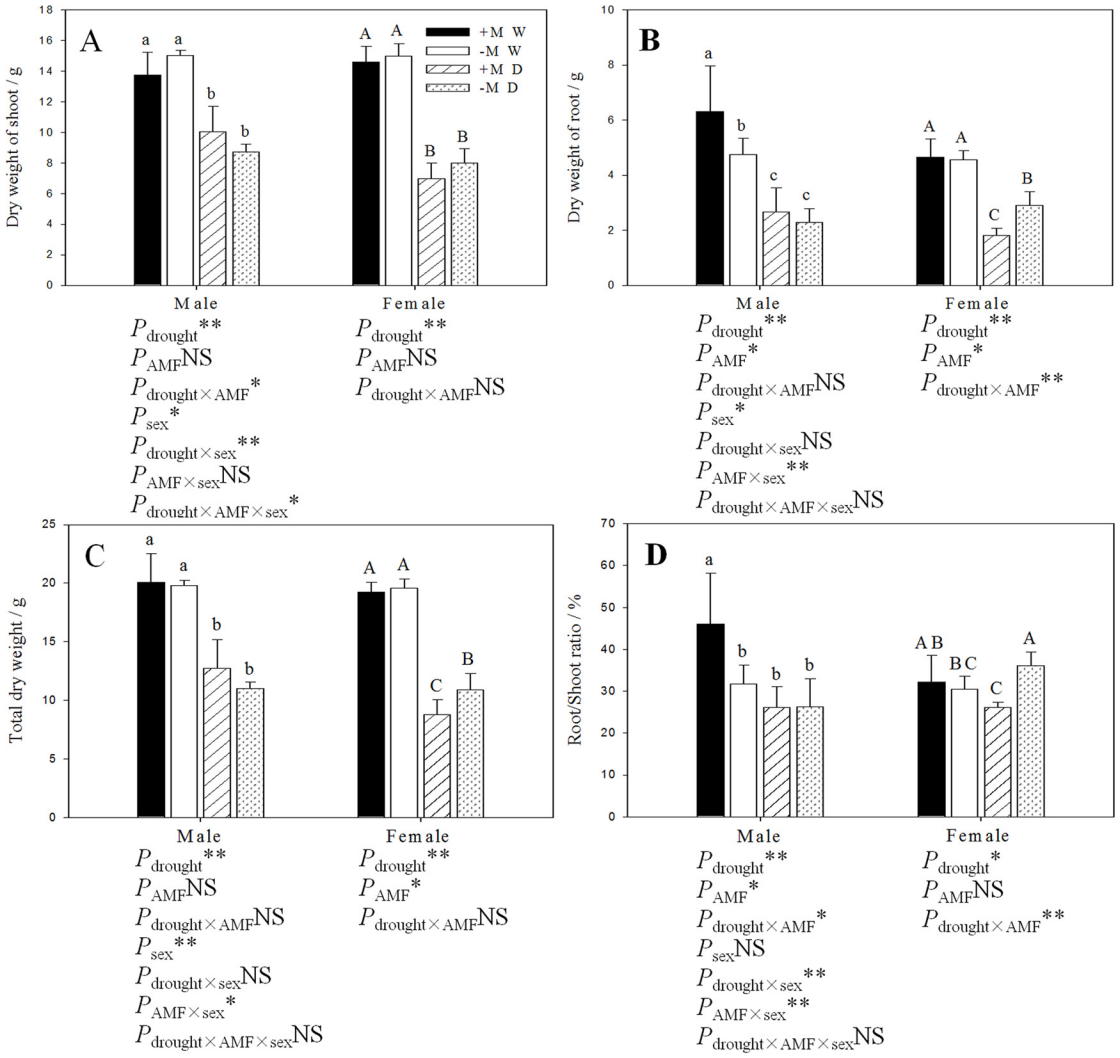
The effect of AM fungi symbiosis on biomass. (A) Dry weight of shoot (DWS); (B) dry weight of root (DWR); (C) total dry weight (DW); (D) root/shoot ratio (RSR) of poplar seedlings under different watering regimes. **Abbreviations**: Treatments: +M W: AM fungi inoculation and well-watered;—M W: non-inoculation and well-watered; +M D: AM fungi inoculation and drought;—M D: non-inoculation and drought. AMF: AM fungi. **Note**: *: significant effect at *P* ≤ 0.05; **: significant effect at *P* ≤ 0.01; NS: no significant effect. Different letters above the error bar indicate a significant difference at *P* ≤ 0.05.

Compared with the well-watered seedlings, the dry weight of the shoot (DWS), the dry weight of the root (DWR) and the total dry weight (DW) of male and female seedlings that had been subjected to the drought treatment were significantly lower. The DWS was not significantly affected by the inoculation treatment in either sex or under either watering regime (Fig 2A). Furthermore, females grown under drought conditions and that had received the inoculation treatment had significantly lower DWR and DW than females that had not received the inoculation treatment (Fig 2B and 2C). Male seedlings grown under well-watered conditions and that had formed an AM fungal symbiosis had a significantly increased root/shoot ratio (RSR) compared with male seedlings grown under drought conditions or that had not formed an AM fungal symbiosis, whereas the RSR of females grown under drought conditions that had formed an AM fungal symbiosis was lower than of females that had not formed an AM fungal symbiosis or that had been grown under well watered conditions (Fig 2D). Three-way ANOVAs indicated that DWR, DWS and DW were significantly affected by sex, and that DWS and RSR were significantly affected by the interaction of drought × sex, and that DWR, DW, and RSR were significantly affected by the interaction of sex × AM fungi, whereas only DWS was obviously affected by the interaction of all three factors.

### Root morphology

Root morphology measurements were obtained from six randomly selected seedlings in each treatment (Table 3). All seedlings grown under drought conditions showed significantly less root growth and development (i.e. reduced RL, RV, RSA and RTN) compared with seedlings grown under well-watered conditions. Male seedlings that had received the AM fungi inoculation treatment had significantly increased RL (well-watered and water-stressed: 5.20% and 102.24%, respectively), RV (13.22% and 79.03%) and RTN (28.42% and 30.49%); however, females that had received the AM fungi inoculation treatment had significantly reduced RV (4.98% and 23.99%) and RTN (30.6% and 30.36%) compared with other female seedlings. Males inoculated with AM fungi and grown under drought conditions had significant advantages over non-inoculated seedlings in terms of RSA. Furthermore, the RL and RAD of females and the RL, RSA, RTN and RAD of males were significantly affected by drought × AM fungi. Three-way ANOVAs showed that males significantly differed from females in terms of root growth except for RSA, and RV and RTN were significantly affected by drought × sex and AM fungi × sex treatment, whereas RL was only significantly affected by AM fungi × sex treatment. Furthermore, drought × sex × AM fungi treatment had a significant effect on RSA, RTN and RAD.

**Table 3:** Effect of AM fungi on root morphology of *P*. *cathayana* males and females under different watering regimes.

Treatment			Root length (cm)	Root volume (cm^3^)	Root surface area (cm^2^)	Root tip number	Root average diameter (mm)
Male	W	+M	22,459.91±1100.48^a^	30.57±0.54^a^	2684.91±143.09^a^	67,244.33±1168.22^a^	0.38±0.01^b^
		–M	21,349.37±1142.98^a^	27.00±1.42^b^	2727.96±473.70^a^	52,364.67±2177.33^b^	0.43±0.00^a^
	D	+M	8714.47±497.88^b^	12.12±1.23^c^	1387.58±308.80^b^	21,659.67±2356.87^c^	0.40±0.03^ab^
		–M	4309.01±393.58^c^	6.77±1.37^d^	573.82±43.24^c^	16,598.67±1312.09^d^	0.37±0.01^b^
*P*_AMF_			**	**	NS	**	NS
*P*_drought_			**	**	**	**	NS
*P*_drought×AMF_			**	NS	*	**	**
Female	W	+M	17,395.50±2016.30^b^	33.19±0.75^b^	2570.03±200.84^a^	73,929.67±4806.92^b^	0.48±0.04^a^
		–M	20,719.47±2472.53^a^	34.93±0.67^a^	2606.97±209.17^a^	106,532.00±19513.38^a^	0.39±0.01^c^
	D	+M	7367.52±628.10^c^	6.37±0.90^d^	721.07±119.61^b^	26,158.00±3667.09^c^	0.38±0.00^c^
		–M	4325.78±855.81^c^	8.38±0.86^c^	719.69±51.67^b^	37,559.67±4939.88^c^	0.43±0.01^b^
*P*_AMF_			NS	**	NS	**	NS
*P*_drought_			**	**	**	**	NS
*P*_drought×AMF_			*	NS	NS	NS	**
*P*_sex_			**	**	NS	**	*
*P*_drought×sex_			NS	**	NS	*	NS
*P*_AMF×sex_			*	**	NS	**	NS
*P*_drought×sex×AMF_			NS	NS	*	*	**

**Abbreviations**: +M: AM fungi inoculation;—M: non-inoculation; W: well-watered; D: drought; AMF: AM fungi.

**Note**:

*: significant effect at 0.01≤ *P* ≤ 0.05;

**: significant effect at *P* ≤ 0.01;

NS: no significant effect. The data are means ± SD (n = 6). Different lowercase letters (a, b, c, d) indicate a significant difference at *P* ≤ 0.05.

### O_2_^−^and H_2_O_2_ content

**Fig 3:**
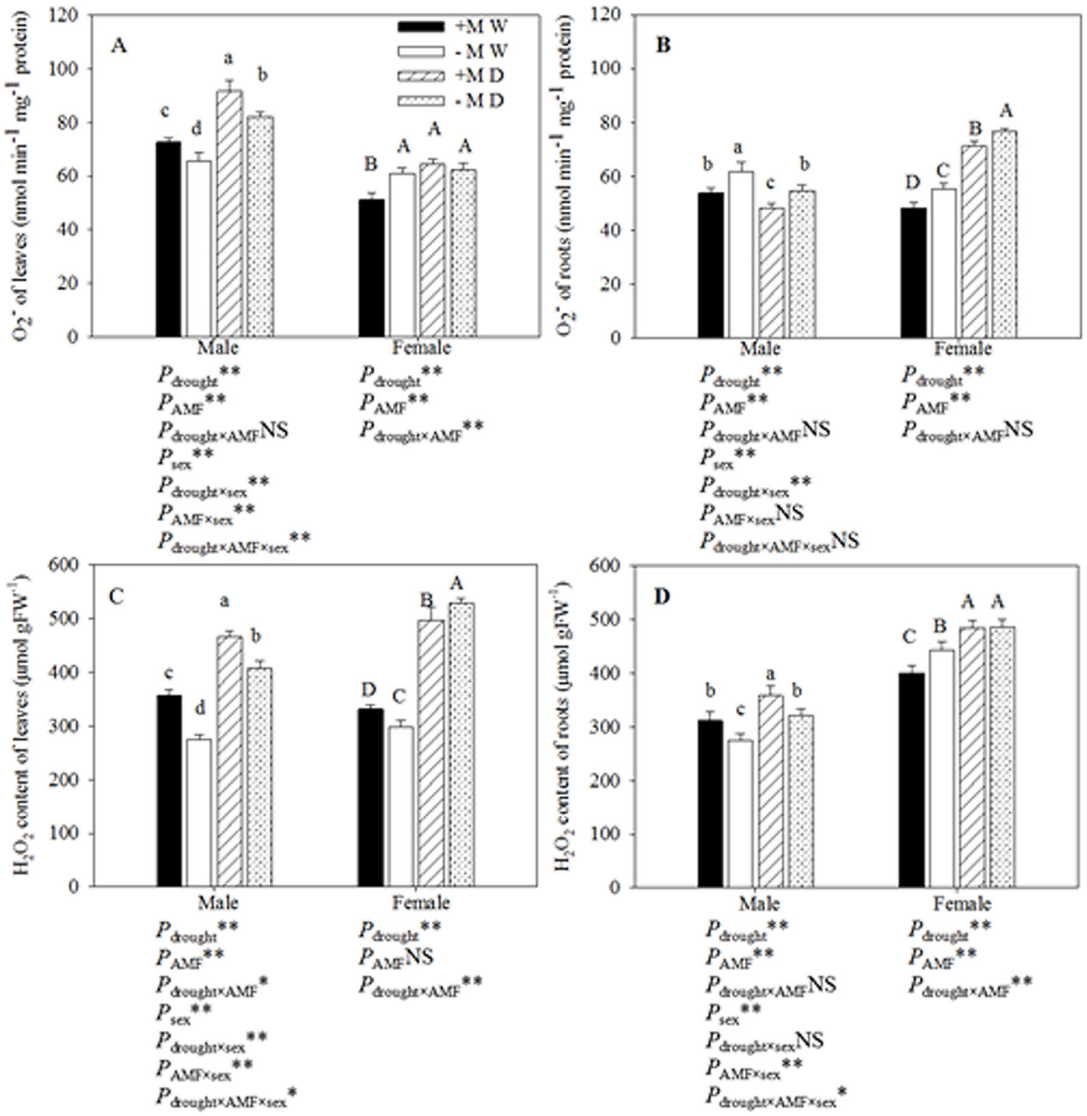
The effect of AM fungal symbiosis on the O_2_– (A, B) and H_2_O_2_ content (C, D) of leaves and roots under different conditions. **Abbreviations**: Treatments: +M W: AM fungi inoculation and well-watered;—M W: non-inoculation and well-watered; +M D: AM fungi inoculation and drought;—M D: non-inoculation and drought; AMF: AM fungi. **Note**: *: significant effect at *P* ≤ 0.05; **: significant effect at *P* ≤ 0.01; NS: no significant effect. Different letters above the error bar indicate a significant difference at *P* ≤ 0.05.

Under drought conditions the O_2_^−^ content in the roots of female seedlings and in the leaves of males, and the H_2_O_2_ content of the leaves of female and male seedlings and the roots of female seedlings was significantly higher than that of seedlings grown under well-watered conditions (Fig 3). However, the O_2_^−^ content of the roots of males significantly decreased under drought conditions. Seedlings that had been inoculated with AM fungi had significantly lower O_2_^−^ content than the non-inoculated seedlings, except in the leaves of males, which had significantly higher O_2_^−^ content; however, seedlings that had been inoculated with AM fungi showed significantly higher H_2_O_2_ content than the non-inoculated seedlings except in the roots of females. Furthermore, the H_2_O_2_ content of males was significantly lower than that of females under all treatment conditions. Three-way ANOVAs indicated that the O_2_^−^ and H_2_O_2_ content in leaves were significantly affected by sex, and the interaction of drought × sex, AM fungi × sex and all three factors. The O_2_^−^ content of roots was significantly affected by sex and the interaction of drought × sex. The H_2_O_2_ content of roots was significantly affected by sex, and the interaction of AM fungi × sex and all three factors.

### POD activity

**Fig 4:**
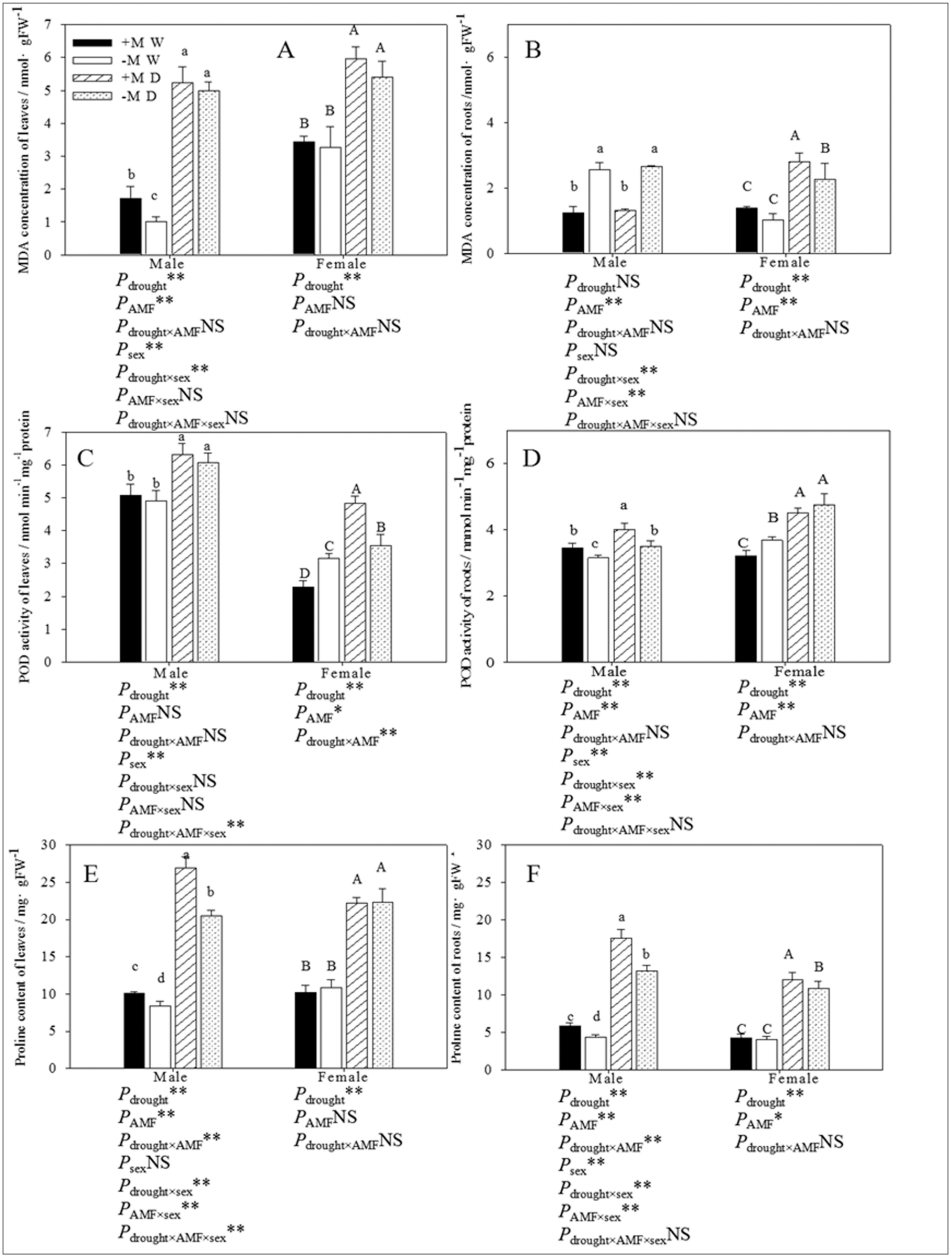
The effect of AM fungal symbiosis on the MDA concentration (A, B), POD activity (C, D) and proline content (E, F) of leaves and roots under different conditions. **Abbreviations**: Treatments: +M W: AM fungi inoculation and well-watered;—M W: non-inoculation and well-watered; +M D: AM fungi inoculation and drought;—M D: non-inoculation and drought; AMF: AM fungi. **Note**: *: significant effect at *P* ≤ 0.05; **: significant effect at *P* ≤ 0.01; NS: no significant effect. Different letters above the error bar indicate a significant difference at *P* ≤ 0.05.

POD activity in each sex showed a similar trend to that of O_2_^−^ and H_2_O_2_ content. POD activity in the leaves of poplar seedlings grown under drought conditions was significantly higher than that in the well-watered seedlings: POD activity in inoculated and non-inoculated males increased by 24.40% and 23.62%, respectively, and in females increased by 112.28% and 12.03%, respectively (Fig 4C and 4D). AM fungi had a different impact on the POD activity of females depending on the watering regime: POD activity increased significantly under well-watered conditions but decreased under drought conditions. In each treatment, the POD activity in the leaves of males was significantly higher than that in females. Three-way ANOVA indicated that there was a significant difference between the POD activity in the leaves of male and female seedlings (*P* ≤ 0.05). Furthermore, POD activity in the leaves of females was significantly affected by drought × inoculation treatment, and was significantly affected by the sex × drought × inoculation treatment.

Drought also promoted POD activity in the roots by 15.92% and 11.06% in males, and by 40.36% and 28.87% in females that received the inoculation and non-inoculation treatments, respectively. Although POD activity in the roots of male seedlings was significantly higher in seedlings that received the inoculation treatment, POD activity was significantly lower in the roots of inoculated female seedlings under well-watered conditions, but was only slightly affected under drought conditions. Three-way ANOVA showed that both sexes differed significantly with regard to the POD activity in their roots, which was also significantly affected by the drought × sex treatment and the inoculation × sex treatment (*P* ≤ 0.01).

### MDA concentration

Under drought conditions the concentration of MDA in the leaves was 67.30% and 79.92% higher in concentrate inoculated and non-inoculated males, and 42.52% and 39.15% higher in inoculated and non-inoculated females than those under well-watered conditions, respectively (Fig 4A and 4B). However, under drought conditions, the concentration of MDA in the roots was only significantly higher than under well-conditions in female seedlings (104.38% and 122.55% in inoculated and non-inoculated females, respectively. Apart from the leaves of males, seedlings that received the inoculation treatment had a significantly higher concentration of MDA in the leaves and roots compared with non-inoculated seedlings. The MDA concentration in the leaves of inoculated males was higher than that in non-inoculated seedlings under both watering conditions. Inoculated seedlings that received the well-watered treatment had a 5.02% and 108.13% higher MDA concentration in the leaves and roots of males, respectively, and a 10.39% and 34.31% higher concentration in the leaves and roots of females, respectively, compared with the uninoculated seedlings. Inoculated seedlings that were subjected to the drought treatment had a 71% and 100.76% higher MDA concentration in the leaves and roots of males, respectively, and 4.27% and 23.35% higher concentration in the leaves and roots of females, respectively, compared with the non-inoculated seedlings. Statistical analysis showed that the MDA concentration in leaves was significantly affected by sex × drought, and that the MDA concentration in roots was significantly affected by sex × drought and by sex × inoculation treatment.

### Proline content

The proline content in the roots of males and females was significantly impacted by drought and AM fungi (Fig 4E and 4F). The proline content in the leaves of males under drought conditions was significantly higher than in seedlings under well-watered conditions in the same inoculated treatments (166.67% and 143.25% in inoculated and non-inoculated males, respectively). However, the proline content of leaves in inoculated and non-inoculated female seedlings was not significantly different, but drought significantly enhanced those (117.34% and 105.66% inoculated and non-inoculated females, respectively). Three-way ANOVA showed that the proline content of leaves was significantly affected by drought × sex, AM fungi × sex and drought × AM fungi × sex, whereas the proline content of roots was significantly affected by drought × sex and AM fungi × sex.

### Correlation analysis

The results of the correlation analysis are shown in Table 4. Significant correlations were observed between most indicators. In particular, apart from H_2_O_2_ content of roots, the MDA concentration in leaves had a significant negative correlation with other indicators, whereas proline and H_2_O_2_ content of leaves were significantly positive with most indicators. In leaves, apart from MDA concentration, all the interactions between indicators of leaves were significantly positive except the interaction of POD activity and H_2_O_2_ content. In roots, all the indicators, other than the interaction of proline content, POD activity and O_2_^−^ and H_2_O_2_ content, had a significantly positive interaction with each other.

**Table 4:** Correlation analysis of MDA concentration, proline content, POD activity, and O_2_^−^ and H_2_O_2_ content of leaves and roots.

Factor		Leaves					Roots				
		MDA	Proline content	POD activity	O_2_^−^	H_2_O_2_ content	MDA	Proline content	POD activity	O_2_^−^	H_2_O_2_ content
Leaves	MDA	1									
	Proline content	–0.837**	1								
	POD activity	–0.721**	0.492**	1							
	O_2_^−^ content	–0.752**	0.574**	0.894**	1						
	H_2_O_2_ content	–0.627**	0.879**	0.263	0.299*	1					
Roots	MDA	–0.282	0.569**	0.371**	0.351*	0.703**	1				
	Proline content	–0.890**	0.946**	0.664**	0.738**	0.783**	0.544**	1			
	POD activity	–0.377**	0.694**	0.030	0.070	0.846**	0.543**	0.528**	1		
	O_2_^−^ content	–0.088	0.264	–0.108	–0.299*	0.519**	0.261	0.089	0.692**	1	
	H_2_O_2_ content	0.031	0.363*	–0.498**	–0.421**	0.551**	0.241	0.123	0.741**	0.522**	1

**Note**:

*: significant effect at 0.01≤ *P* ≤ 0.05;

**: significant effect at *P* ≤ 0.01.

## Discussion

### Shoot and root biomass measurement

Generally, the formation of an AM fungal symbiosis results in an increase in plant biomass accumulation and an altered root/shoot ratio [23]. In our study, the responses to AM fungal formation differed between males and females, and AM fungal symbiosis only had a marked positive effect on the root biomass of males when grown under well-watered conditions. AM fungal symbiosis had a positive effect on male poplar growth but had no impact on the growth of females. The response of males to AM fungi supports most previous studies in trees showing that AM fungi enhances the growth and photosynthesis of the tree, which is reflected by an increase in biomass accumulation [11,23]. However, some studies have reported that formation of an AM fungal symbiosis had either a negative impact or no impact on the plant host [33], which supports our findings that AM fungal formation in females had limited impact. Orlowska et al. [34] found inoculation of *R*. *intraradices* had a lower survival rate and shoot biomass accumulation, but a higher root biomass accumulation than other AM fungi. However, Liu et al. [35] suggested a positive effect of *R*. *intraradices* on poplar biomass and bioenergy accumulation. These researches indicated that a potentially complex effect of *R*. *intraradices* on host plants, which may caused the gender difference in this study. It has been well documented that *P*. *cathayana* males are able to tolerate stressful environments, such as drought [5], soil nutrition deficiency [4] and pathogenic symbiosis [30] better than female plants. A possible explanation is that a nutrient deficiency in the soil matrix as a result of autoclaving and the need to supply the AM fungus with carbon could be more disadvantageous for pot-grown *P*. *cathayana* females in terms of root and shoot growth than for males.

### Shoot and root morphology measurement

The drought response involves morphological, physiological, and biochemical changes. Our study indicated that water stress caused a significant decrease in GSL, GGD and LA in *P*. *cathayana*, which supports similar findings in *Brachypodium distachyon* [36], and *Sorghum bicolor* L. [37]. We also showed that *P*. *cathayana* seedlings showed different morphological, physiological and biochemical responses to drought and inoculation with AM fungi depending on their sex. Well-watered females showed higher GSL than males without AM fungi, but lower GSL than males that had formed an AM fungal symbiosis, which suggests that AM fungal symbiosis has a different affect on male and female seedlings, which supports the findings of Lu et al. [25]. We suggested that these differences are due to different gender specializations [5].

Photosynthesis enables the plant to assimilate CO_2_, which can be used for plant growth. Water is one of the main factors in photosynthesis and, hence, water limitation affects plant growth. This supports our findings that the shoot and root dry weights of both sexes grown under well-watered conditions were significantly higher than those grown under drought conditions.

Drought is one of the harshest constraints limiting root growth and ecosystem productivity [38]. Previous studies have suggested that *P*. *cathayana* males were able to tolerate drought conditions better than females [5,8]; however, the influence of AM fungi has not previously been investigated. Our study showed that although drought significantly limited RL, RV, RSA and RTN in female and male poplars, males that had formed an AM fungal symbiosis had significantly greater RL, RV and RAD than the females that had formed an AM fungal symbiosis and, hence, the males showed better drought tolerance than females. A better and thicker root system should enable more water to be taken up from lower soil layers and from a wider area, improving water uptake under water-limiting conditions and, hence, a better and thicker root system is likely to be a key factor contributing to the drought stress resistance of plants [39]. Improved water uptake also enables the plant to maintain a good water potential, which has been shown to have a positive effect on growth under drought stress [40]. Furthermore, AM fungi hyphae are able to penetrate the soil pores that are inaccessible to roots, making water in the soil more accessible to the roots [41]. This may explain why males that formed an AM fungal symbiosis were able to tolerate drought significantly better than non-inoculated males.

### ROS, antioxidant system and osmotic adjustment of shoots and roots

Better and thicker roots result in the uptake of more water, which results in improved oxidative protection and a stable osmotic balance [42]. ROS, such as O_2_^−^ and H_2_O_2_, are kept in dynamic balance. Under conditions of water deficit, the balance is broken, leading to more ROS, which cause oxidative damage [43]. In keeping with these previous findings, in our study, the O_2_^−^ and H_2_O_2_ contents were significantly higher in seedlings grown under drought conditions. Proline, an indicator of the extent of lipid peroxidation caused by oxidative stress and an osmolyte for plant osmotic adjustment, accumulates in higher plants in respinse to water deficit [15], which supports the increase in proline shown in our study under drought conditions.

AM fungal formation contributes to the production of scavenging peroxyl radicals, buffering cellular radical potential [44], and a more powerful ROS-scavenging system [45]. Plants that form a symbiotic relationship with an AM fungus have been shown to have lower lipid peroxidation and higher antioxidant enzyme activity in leaves [46], which supports our findings that drought significantly enhanced MDA concentration, proline content and the POD activity of shoots in both sexes. The proline content and POD activity of roots were significantly stimulated by water stress. However, the MDA concentration of roots in males was significantly affected by AM fungi rather than by drought: inoculated roots had a significantly lower MDA concentration in the roots of males, which agreed with the findings of Wu et al. [47]. Furthermore, AM fungi had a greater impact on the roots than on the shoots. We hypothesize that the AM fungal symbiosis mainly affects the function of the root rather than the shoot given that the ecological niche of AM fungi is in the plant root and rhizosphere. This idea is supported by the finding that the MDA concentration in shoots showed similar trends in both sexes, suggesting that the formation of AM fungi chiefly affected the below-ground structures. Plants that formed a symbiosis with AM fungi had increased photosynthetic product, and female seedlings were more sensitive to drought and suffered more oxidative damage, reflected by the higher concentration of MDA.

In our study, proline content significantly increased under drought conditions, which has been well-documented by previous studies [15]. However, the proline content in leaves was higher than that in roots, which suggests that AM fungi assisted *P*. *cathayana* in accumulating proline for sub-cellular stability. Furthermore, the accumulation of proline is a highly regulated process involving a set of protein kinases and is ubiquitous for stress tolerance [48]. However, the POD activity of roots showed a distinct phenomenon: plants that formed a symbiotic relationship with the AM fungus showed slightly increased POD activity in male seedlings but slightly decreased POD activity in female seedlings. Given that females are more sensitive to environmental stresses [5], the increased accumulation of proline and POD activity in inoculated *P*. *cathayana* seedlings suggests that inoculating seedlings with an AM fungus may have the potential to activate these protein kinases for plant drought tolerance, but further investigation is needed.

Our research revealed that certain morphological structures and physiological functions of shoots and roots of male and female *P*. *cathayana* were significantly affected by drought stress and AM fungi inoculation. Drought stress simultaneously significantly limited the morphological development of shoots and roots of both sexes. However, although AM fungal symbiosis had a significant positive effect on males, it had only a slight or no effect on females. Drought also limited biomass stimulation. AM fungi only had a significant positive impact on root biomass and on the root/shoot ratio of males under well-watered conditions, and only had a significant negative impact on root biomass, total biomass and on the root/shoot ratio of females grown under drought conditions. AM fungi affected the antioxidant system and osmotic adjustment system of shoots and roots in different ways. AM fungi had more obvious effects on the roots than on the shoots, reflected by the finding that POD activity and proline content were significantly affected by drought and AM fungi. Therefore, AM fungi plays a more effective role in facilitating drought-induced protection in males than in females, including positive effects on the morphological structure and physiological function and development of shoots and roots.
