# Correction: Sex-Related Responses of *Populus cathayana* Shoots and Roots to AM Fungi and Drought Stress

**DOI:** 10.1371/journal.pone.0142356

**Published:** 2015-11-03

**Authors:** The PLOS ONE Staff

The first author, Zhen Li, should not have been noted as being a corresponding author of this work. Ming Tang (tangm@nwsuaf.edu.cn) is the only corresponding author. The publisher apologizes for the error.
